# Spatial analyses of two color polymorphisms in an alpine grasshopper reveal a role of small‐scale heterogeneity

**DOI:** 10.1002/ece3.4156

**Published:** 2018-06-27

**Authors:** Petra Dieker, Luisa Beckmann, Julia Teckentrup, Holger Schielzeth

**Affiliations:** ^1^ Department of Evolutionary Biology Bielefeld University Bielefeld Germany; ^2^ Department of Population Ecology Institute of Ecology and Evolution Friedrich Schiller University Jena Jena Germany

**Keywords:** balancing selection, Color polymorphism, habitat heterogeneity, local adaptation, Orthoptera

## Abstract

Discrete color polymorphisms represent a fascinating aspect of intraspecific diversity. Color morph ratios often vary clinally, but in some cases, there are no marked clines and mixes of different morphs occur at appreciable frequencies in most populations. This poses the questions of how polymorphisms are maintained. We here study the spatial and temporal distribution of a very conspicuous color polymorphism in the club‐legged grasshopper *Gomphocerus sibiricus*. The species occurs in a green and a nongreen (predominately brown) morph, a green–brown polymorphism that is common among Orthopteran insects. We sampled color morph ratios at 42 sites across the alpine range of the species and related color morph ratios to local habitat parameters and climatic conditions. Green morphs occurred in both sexes, and their morph ratios were highly correlated among sites, suggesting shared control of the polymorphism in females and males. We found that in at least 40 of 42 sites green and brown morphs co‐occurred with proportions of green ranging from 0% to 70% with significant spatial heterogeneity. The proportion of green individuals tended to increase with decreasing summer and winter precipitations. Nongreen individuals can be further distinguished into brown and pied individuals, and again, this polymorphism is shared with other grasshopper species. We found pied individuals at all sites with proportions ranging from 3% to 75%, with slight, but significant variation between years. Pied morphs show a clinal increase in frequency from east to west and decreased with altitude and lower temperatures and were more common on grazed sites. The results suggest that both small‐scale and large‐scale spatial heterogeneity affects color morph ratios. The almost universal co‐occurrence of all three color morphs argues against strong effects of genetic drift. Instead, the data suggest that small‐scale migration–selection balance and/or local balancing selection maintain populations polymorphic.

## 1 INTRODUCTION

Balanced intraspecific color polymorphisms have fascinated researchers for a long time, because they demand eco‐evolutionary explanations for how polymorphisms are maintained in the long run (Fisher, 1930; Ford, 1965; Huxley, 1955; Svensson, 2017). The mechanisms that maintain populations polymorphic are, however, often unexplored and likely differ between species. The insect order Orthoptera—the crickets, bush‐crickets and grasshoppers—is particularly remarkable because it represents large, phylogenetically old clade in which various polymorphisms, including a conspicuous green–brown polymorphism, are shared among species (Dearn, 1990; Rowell, 1971). Among the Central and Western European Orthoptera, for example, about 20% of the species are known to be green–brown polymorphic (Bellmann, 2006; Bellmann & Luquet, 2009) and this color polymorphism occurs in both suborders, Ensifera and Caelifera, that have diverged about 200 Mya (Misof et al., 2014). Even some stick insects, Phasmatodea, and matids, Mantodea, two groups that have diverged from the Orthoptera about 250 Mya (Misof et al., 2014), are green–brown polymorphic. The old age of the clade and the immense number of green–brown polymorphic species, interspersed with some exclusively brown and some exclusively green taxa, makes Orthopterans particularly suitable for studying the mechanisms that maintain balanced color polymorphisms.

Color polymorphisms often have a genetic basis, and the rarest morph appears too frequent as to be explained solely by recurrent mutation (Fisher, 1930; Huxley, 1955). Genetically based phenotypic polymorphisms are necessarily labile to loss by directional selection favoring the most adapted morph and to random loss by genetic drift. So which mechanisms maintain populations polymorphic? Polygenic traits can remain polymorphic in mutation–selection–drift balance when selection at individual loci is weak and the mutational target is large (Bürger & Lande, 1994; Bürger, Wagner, & Stettinger, 1989), but, as we argue below, we consider it unlikely that the green–brown color polymorphisms is a highly polygenic trait. Temporally fluctuating selection may help to maintain some polymorphisms, even though fluctuating selection is susceptible to overshooting (Bell, 2010; Sasaki & Ellner, 1997). More efficient may be spatially heterogeneous selections with gene flow that can maintain populations polymorphic in migration–selection balance (Yeaman & Whitlock, 2011). Finally, selection itself may favor polymorphisms if either different fitness components are involved in selective trade‐off and lead to net disruptive selection or if selection is negative frequency‐dependent (Fitzpatrick, Feder, Rowe, & Sokolowski, 2007; Gigord, Macnair, & Smithson, 2001). Not all cases of discrete phenotypes need to be genetically controlled, and some may be due to phenotypic plasticity (West Eberhard, 2003). This, however, only shifts the question to how selection favors the maintenance of phenotypic plasticity in the light of the added costs that are likely to occur when two alternative options need to be maintained.

The green color in Orthoptera is formed by tetrapyrroles, in particular biliverdin (Fuzeau‐Braesch, 1972). The genetic pathway to the production of a green skin thus needs to include functional metabolism, transport, and deposition of the green pigments in the developing skin prior to regular molting between nymphal instars and finally into the adult insect (Shamim, Ranjan, Pandey, & Ramani, 2014). As we consider green–brown polymorphisms that are discrete by state rather than gradual variation along a continuum, the pathway(s) seem(s) to be switched off in brown individuals that lack green colors entirely. Loss‐of‐function mutations are likely to occur more frequently than gain‐of‐function mutation that restore the genetic pathway (Behe, 2010). We therefore argue that the discrete nature of the polymorphism, the apparently simple genetic inheritance in green–brown polymorphic stick insects (Comeault, Carvalho, Dennis, Soria‐Carrasco, & Nosil, 2016) and the presumably relatively simple biochemical pathway, makes polygenic inheritance and thus mutation–selection–drift balance less likely in the case of the green–brown polymorphism in Orthopterans. Formal genetic studies are, however, required to verify this interpretation.

Temporal and spatially variable selections are possible mechanisms even for discrete traits that are controlled by few genes. Fluctuating selection has been claimed to be common in natural populations (Siepielski, DiBattista, & Carlson, 2009), although this conclusion has been challenged (Morrissey & Hadfield, 2012). But even if selection is fluctuating, the system needs to be very fine‐tuned with sufficiently strong selection to counter genetic drift effects and regular switches in the sign of selection in order to protect populations against accidental loss of one of the morphs. While such systems may occur, it is not trivial to find a situation in which selection varies not only in magnitude, but also in direction on a regular basis. More promising for the long‐term maintenance of polymorphisms seems spatially heterogeneous selection with gene flow or systematic temporal changes in selection induced by negative frequency dependency.

In some species of Orthoptera, the green–brown polymorphism seems to be under environmental control with background color of the habitat and moisture being the main determinants during nymphal development (Dearn, 1990; Rowell, 1971). However, this is not universally true and, in some species, including the species that we study here, background coloration does not affect color morph development (Valverde & Schielzeth, 2015). Notably the green–brown polymorphism that we address here is different from the phase polymorphism in some locusts (Song, 2011). The phase polymorphism is triggered by density and is mediated via juvenile hormone that coordinates changes in color along with changes in morphology, physiology, and behavior (Tanaka, Harano, Nishide, & Sugahara, 2016). In phase polymorphism systems, it is difficult to distinguish those different components. Our color polymorphism system, in contrast, suggests that color itself is under selection.

**Figure 1:**
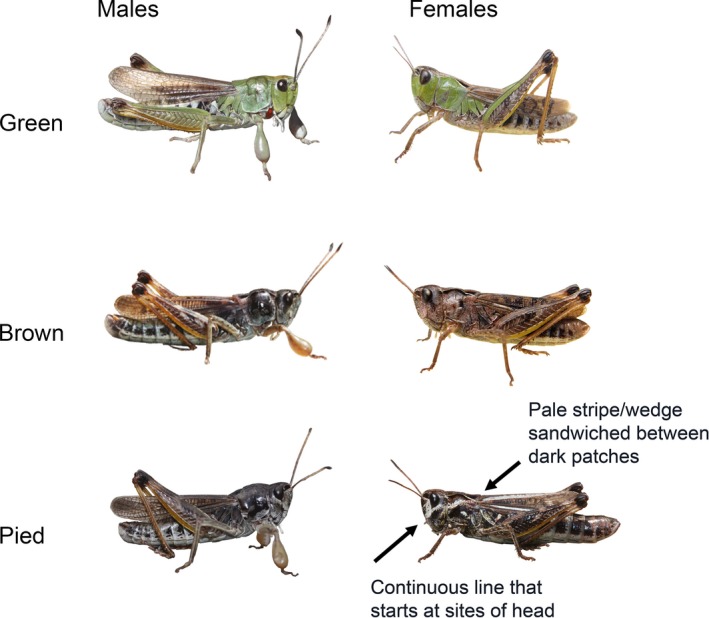
Typical examples of the three color morphs in males and females. Only green morphs show green color. Pied morphs are distinguished from brown morphs by the characteristic pale transverse stripe across the sides of the head and the pronotum. Brown morphs are very variable in darkness but are overall rather uniformly colored. This particular green male has an ecoparasitic mite attached near the mandibles

We here study the club‐legged grasshopper *Gomphocerus sibiricus* (Linnaeus 1767), an alpine ground‐dwelling species that displays a striking green–brown color polymorphism in both sexes. In most populations in the European Alps, the brown morphs are more abundant than the green morphs (see below). We combine large‐scale with small‐scale spatial and temporal sampling that allows us to address the evolutionary ecology of the color polymorphism in this species. We predict that color morph ratios are spatially variable and predictable by environmental conditions if selection for local adaption contributes to the maintenance of color polymorphisms in this species. Besides the striking green–brown polymorphism, the species also features a distinctive pied morph that, like brown morphs, lacks any green pigments, but is characterized by a black‐and‐white contrast on head and pronotum (Figure 1). Pied morphs occur at lower numbers, but are also widespread and the questions about the maintenance of the polymorphism applies equally to pied morphs.

## 2 MATERIAL AND METHODS

### 2.1 Sampling sites

We sampled 42 sites from across large parts of the alpine range of the club‐legged grasshopper from eastern Austria to southwestern Switzerland, covering most of the altitudinal range of the species (Table S1). We identified target *regions* (17 regions in total), operationally defined as mountain areas without division by major valleys and inhabitable mountain ridges, and aimed to sample two to three *sites* per region (with some variation) that differed in habitat, in particular altitude, aspect, and/or vegetation cover. Nearest‐neighbor distances were 8–92 km (mean 51 km) among regions and 0.2–23 km (mean 5 km) among sites within regions. From each site, we aimed to sample 50–100 individuals and to score them for their color morph. A priori power analysis shows that with 50 (100) individuals, the expected standard error is about ±0.067 (±0.047) for estimated proportions in the range of minor morph frequencies around 0.33.

At one of the sites, a large SW‐facing slope in Central Austria (site Albitzen I/Heiligenblut), we sampled at six *locations* in two consecutive years in order to estimate the local spatial and temporal repeatability of color morph ratios. This site was chosen, because the habitat was visually rather homogenous without obvious barriers to dispersal and we hence expect stable morph ratios among the six nonoverlapping sampling locations. Locations were separated by about 50 m. Any remaining heterogeneity beyond sampling variance may be attributed to fine‐scale microhabitat heterogeneity.

We refer to variation among regions as *large‐scale variation*, as it involves a scale of multiple kilometers and populations that are separated by dispersal barriers. We refer to variation among sites within regions as *small‐scale variation*, as it involves a scale of a few hundred meters to a few kilometers (sometimes a few dozen kilometers) among sites that are not separated by major dispersal barriers, but that differ in habitat structure. Finally, we refer to variation among locations within sites to *fine‐scale variation*, since it involves a scale of a few dozen meters with no dispersal barriers and no habitat differences.

### 2.2 Field sampling and scoring

Grasshoppers were sampled in July and August in 2014 and 2015 (Table S1), and in August 2016, we resampled the six locations at the site Albitzen I/Heiligenblut (Table S1). Sampling was carried out between late morning and late afternoon under warm conditions when grasshoppers are mostly active. Grasshoppers were detected by slowly walking across the low‐vegetation habitats based on their escape movements. Detected individuals were caught by hand or using small nets. As grasshoppers were detected by motion rather than when sitting still on their natural background, detection was independent of color, albeit not necessarily independent of activity levels. Individuals were temporarily retained in small vials and when sufficient numbers of individuals were caught, they were all scored for their color morphs. This procedure ensured that no individual was recorded twice. Individuals were released on spot after scoring.

All individuals were sexed, and their color morph was recorded. We scored three morphs that we call green, brown, and pied (Figure 1). Green individuals are readily identified by their green pronotal lobes (and sometimes dorsal parts of the pronotum) as well as green frontal part of the head. Individuals darken within a few days after imaginal molting (Valverde & Schielzeth, 2015), and while green females are always immediately recognized, green males can become so dark that the green parts are barely visible in side views. However, the diagnostic green front always reveals even the darkest green males. Importantly, only green morph individuals display green colors. Brown individuals are highly variable greyish, brownish, or blackish, but always lack green tones, and they also lack the characteristic pattern of pied morphs. Pied morphs show distinct black‐and‐white stripes across the sides of the head and the pronotum and typically (particularly in females) show a variable black face mask. Pied males, while being still recognizable as those in the last instar nymphal stages, darken so much as imagoes that they are often indistinguishable from brown morphs. We therefore analyze the prevalence of green morphs using data from both sexes (green vs. brown/pied) and the prevalence of pied morphs for the female sample only (pied vs. brown/green). Along with sex and morph identities, we recorded the extent of the face mask (Figure S1) as well as marked deformations and mite infections.

### 2.3 Habitat characterization

Local habitat characteristics were compiled from three sources. First, we estimated vegetation cover on spot. Specifically, we scored the percentage cover of (i) bare ground, (ii) gravel and stones, (iii) herbal and grassy vegetation, (iv) low‐growing shrubs (*Vaccinium myrtillus*,* V. ulingosum*,* V. vitis‐idaea*,* Arctostaphylos uva‐ursi*), and (v) junipers. Furthermore, we noted whether the habitats were grazed, indicated by presence or feces of cattle. Second, we derived topographical characteristics, that is, elevation and aspect, from a digital elevation model (DEM; spatial resolution of 30 arc‐seconds) (USGS/GLCF 2000) for each site and transformed the aspect data into north–south and east–west component by arcsine and cosine transformations (Leyer & Wesche, 2007). Sampling dates were recorded along with geographic positions (latitude and longitude). Third, we downloaded all 19 BIOCLIM variables from the CHELSA (Climatologies at high resolution for the earth's land surface areas) data (Karger et al., 2017a,2017b) to characterize the general predominant climate at our study sites. The CHELSA data depict downscaled model output temperature and precipitation estimates of the ERA‐Interim climatic reanalysis (spatial resolution of 30 arc‐seconds) and refer to the period 1979–2013 (Karger et al., 2017a,2017b). As BIOCLIM variables are highly correlated among each other, we used principle component analysis to summarize climatic variation in independent dimensions.

As color morphs may differ in their thermal activity, which could lead to differences in catching probabilities, we gathered sunshine duration for the sampling days (estimated in hours) and cloud cover following the Okta classification by estimating how many eighths of the sky were covered by clouds (World Meterological Organization 2008).

### 2.4 Statistical analyses

We fitted generalized linear mixed models (GLMM) with a binomial error distribution and logit link to color morph identities with sex and environmental predictor variables fitted as fixed effects and site and region as random effects in the model. The response was coded as binary (yes/no) rather than aggregated to proportions. The analysis of pied morphs was limited to females, and we therefore omitted sex as a predictor. Variance components are summarized as intraclass correlations (repeatabilities) *R*, which are variance components standardized by the total phenotypic variance on the observed scale (Nakagawa & Schielzeth, 2010). Uncertainties in the estimates of intraclass correlations were quantified by parametric bootstrapping.

We explored specific environmental predictors that may explain spatial variability in morph ratios. Predictors were grouped in three classes: (i) geographic predictors, in particular longitude, latitude, altitude, and aspect; (ii) general climatic conditions based on BIOCLIM data; and (iii) local habitat characteristics recorded in the field. Within each class, we fitted predictors one at a time as well as in combination. When fitting predictors in isolation, we used Benjamini and Hochberg (1995) family‐wise false‐discovery rate control for adjusting *p* values for multiple testing. In the case of the multiple regression, we apply full model test within classes to control for multiple testing as recommended (Forstmeier & Schielzeth, 2011), which in our case involves keeping the random effects and sex as a fixed effect in both the full and the reduced models. We also fitted a model that included all predictors from all classes simultaneously, but this model did not converge due to the unfavorable ratio of predictors to populations sampled. The full model test across all variables should therefore be treated with caution.

All models were fitted in R 3.4.3 using the package lme4 1.1‐12 for fitting mixed‐effects models (Bates, Mächler, Bolker, & Walker, 2015) and the package rptR 0.9.2 for quantifying ratios of variance components (Stoffel, Nakagawa, & Schielzeth, 2017). Furthermore, we used the p.adjust and the prcomp functions form the basic stats package (R Core Team, 2016) for false‐discovery rate control and principle component analysis, respectively.

## 3 RESULTS

We sampled a total of 4,281 individuals from 42 sites across 17 regions in the Swiss and Austrian Alps. Six hundred of these individuals were sampled at six locations (100 individuals each) at a homogenous slope in central Austrian in 2015 and another 318 individuals at the same six locations in 2016 (a wetter and colder year compared to 2015 with overall much lower grasshopper densities). From the remaining sites, we sampled, on average (±*SD*), 80 ± 40 (range 10–154) individuals per site. A total of 2,064 individuals (48%) were females and 2,217 (52%) males.

**Figure 2:**
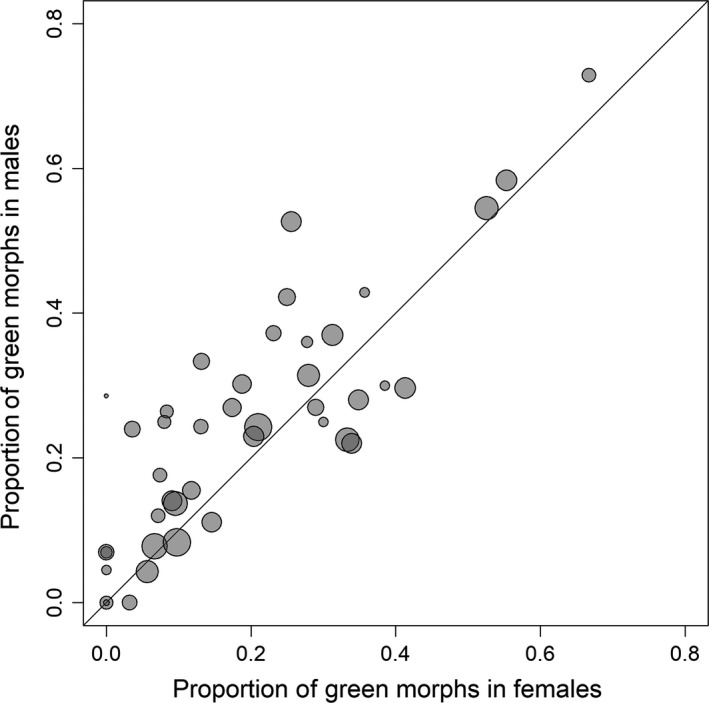
Proportion of green females and green males across all 42 sampling sites with the size of the dots proportional to the geometric mean sample size in the two sexes. The line shows equal proportions in both sexes. For the slope with high levels of local replication and thus for larger sample size (site Albitzen I/Heiligenblut), we selected a single subsample as for the large‐scale spatial analysis

A total of 1,093 individuals were green (24% in females, 27% in males), 2,479 individuals were brown (52% in females, 64% in males), and 709 individuals were pied (25% in females, 9% in males). Besides the obvious deficiency of pied males (due to blurring of the patterns by darkening), there was a slight, but significant excess of green individuals in males (GLMM: b = 0.19 ± 0.07, z = 2.55, *p* = .011). The proportion of green morphs was highly correlated between the two sexes across sites (*r* = .83, t_40_ = 9.32, *p* < 10^−10^, Figure 2).

### 3.1 Fine‐scale spatial and temporal heterogeneity within sites

**Figure 3:**
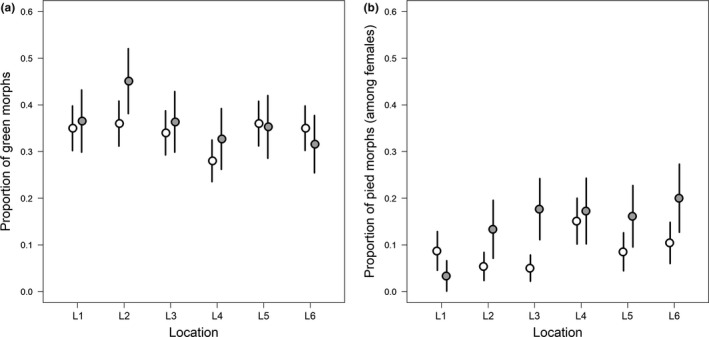
(a) Proportion of green individuals from six locations across a visually homogenous slope at the center of the alpine distribution as sampled in 2015 (open symbols) and 2016 (gray symbols). (b) Proportion of pied morphs among females across the same size location in the same 2 years. Vertical lines show *SE* approximated by resampling

Fine‐scale sampling across six locations on a homogenous mountain slope in Central Austria (i.e., within a single site) resulted in fractions of green morph individuals between 28% and 36% in 2015 and 32% and 45% at the very same locations in 2016 (Figure 3). The average proportion of green morphs across locations (35%) was thus clearly above the average across the remainder of the populations. Green morph ratios did not vary significantly among locations in either year (*R* = .00 ± .01, $\chi_{1}^{2}$ = 0.00, *p* = 1.0 in both years), and there was no significant difference in the proportion of green individuals among years (GLMM: b = 0.09 ± 0.15, z = 0.64, *p* = .52). At the same site, pied morphs occurred at 5%–15% among females in 2015 and 3%–20% in 2016 (Figure 3). The average proportion of pied morphs (6%) is thus clearly below the average across the remainder of the sites. Pied morph ratios did not vary significantly among locations in either year (*R* = .00 ± .01, $\chi_{1}^{2}$ = 0.00, *p* = 1.0 in both years), but there were significantly more pied individuals present in 2016 as compared to 2015 (GLMM: b = 0.59 ± 0.29, z = 2.03, *p* = .042). In order to avoid giving undue weight on the single site with large sample size, we included only one 2015 sample from one of the locations in the following large‐scale analysis (results were not susceptible to which location was chosen).

### 3.2 Smale‐scale and large‐scale spatial heterogeneity among sites and regions

**Figure 4:**
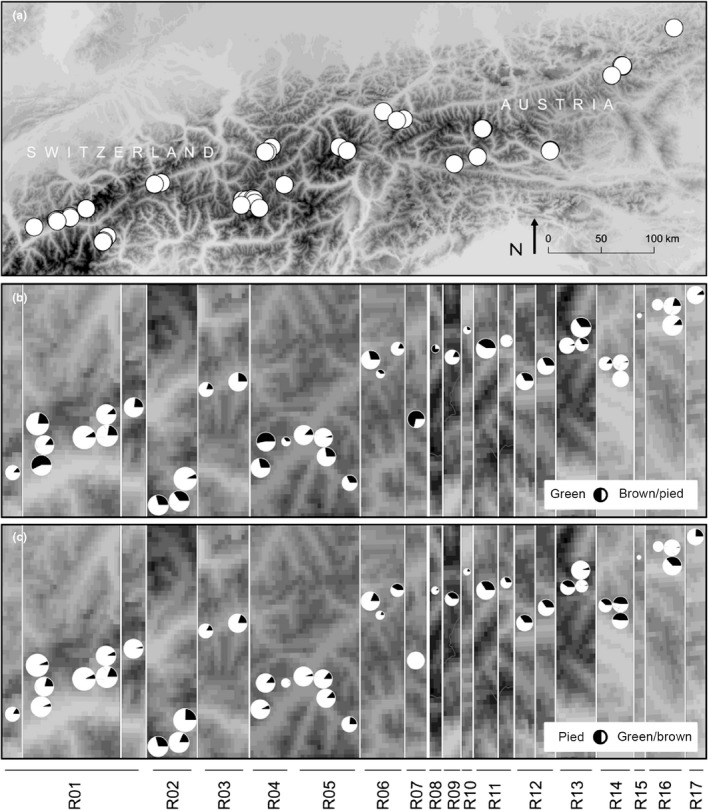
(a) Overview about the 42 sites across the Swiss and Austrian Alps that have been sampled for color morph ratios of *Gomphocerus sibiricus*. (b) Proportion of green and brown/pied individuals across all sites as clustered in 17 regions. (c) Proportion of pied and brown/green individuals indicated by black and white coloration, respective, across all sites as clustered for the same 17 regions. Dot size is proportional to the geometric mean sample size

Green morph ratios varied between 0% at two nearby sites in Eastern Austria (with samples sizes of *N* = 10 and 58 individuals, respectively) and three sites with a majority of green morphs (53%, 58%, and 70%, all *N* ≥ 86, Figure 4). There was no significant heterogeneity among regions (*R* = .033 ± .028, $\chi_{1}^{2}$ = 1.14, *p* = .14), but significant heterogeneity among sites within regions (*R* = .115 ± .036, $\chi_{1}^{2}$ = 191.0, *p* < 10^−40^), so in total about 14% of the variation was among sites. There was no indication that differences were systematically produced by seasonal variability (GLMM: b = 0.01 ± 0.02, z = 0.58, *p* = .56), daytime (b = 0.05 ± 0.07, z = 0.77, *p* = .44), or weather conditions (cloud cover and sunshine duration all |z| < 0.41, *p* > .68).

Pied morph ratios varied between 3% (*N* = 27) and 75% (*N* = 24) plus two sites with 100% pied but very low sample size of females (*N* = 2 and 4). There was significant heterogeneity among regions (*R* = .075 ± .042, $\chi_{1}^{2}$ = 5.8, *p* = .0080) and among sites within regions (*R* = .084 ± .027, $\chi_{1}^{2}$ = 41, *p* < 10^−10^), so in total about 15% of the variation was among sites. There was no indication that differences were systematically produced by seasonal differences, daytime, cloud cover, or sunshine duration (GLMM: all |z| < 1.20, *p* > .23).

### 3.3 Geographic and climatic predictors

The proportion of green individuals did not depend on altitude, latitude, longitude, or aspect (Table 1). The proportion of green individuals tended to increase with PC1 of the BIOCLIM variables, although this effect was not robust to multiple testing control and thus should be treated with caution (Table 1). Precipitation loaded most strongly and negatively on PC1, and the trend was thus toward an increase in green individuals with decreasing precipitation (Table 2).

**Table 1:** Regression slopes of color morph ratios on geographical, climatic, and habitat variables

	Univariate				Multivariate			
	b	z	*p*	p*	b	Z	*p*	FMT
Green morphs								
Geographic parameters								
Altitude	0.07 ± 0.07	0.99	.32	.55	0.06 ± 0.06	0.93	.35	$\chi_{5}^{2}$ = 6.68, *p* = .24
Latitude	−0.81 ± 0.47	−1.73	.08	.24	0.19 ± 0.80	0.24	.81	
Longitude	−0.16 ± 0.08	−1.94	.052	.24	−0.13 ± 0.14	−0.97	.33	
Aspect (sin)	0.02 ± 0.28	0.07	.94	.86	0.04 ± 0.31	0.12	.91	
Aspect (cos)	0.40 ± 0.29	1.35	.17	.24	0.45 ± 0.30	1.49	.14	
Climatic parameters								
Climate PC1	0.15 ± 0.06	2.42	**.015**	.11	0.14 ± 0.05	2.63	**.0084**	$\chi_{4}^{2}$ = 7.91, *p* = .095
Climate PC2	0.12 ± 0.09	1.35	.18	.38	0.11 ± 0.07	1.50	.13	
Climate PC3	−0.01 ± 0.09	−0.06	.95	.95	0.01 ± 0.07	0.08	.94	
Climate PC4	−0.02 ± 0.16	−0.13	.90	.95	−0.04 ± 0.14	−0.26	.80	
Habitat parameters								
Bare ground	0.08 ± 0.03	2.88	**.004**	.06	0.05 ± 0.04	1.20	.23	$\chi_{6}^{2}$ = 12.23, *p* = .057
Shrub cover	−0.01 ± 0.01	−0.84	.40	.54	−0.02 ± 0.02	−1.07	.29	
Stone cover	−0.01 ± 0.02	−0.37	.71	.86	−0.02 ± 0.03	−0.66	.51	
Vegetation cover	−0.01 ± 0.01	−0.88	.37	.55	−0.02 ± 0.02	−1.07	.29	
Juniperus cover	0.02 ± 0.02	0.94	.35	.55	0.00 ± 0.03	−0.10	.92	
Grazing	−0.58 ± 0.33	−1.79	.07	.24	−0.36 ± 0.31	−1.15	.25	
Pied morphs								
Geographic parameters								
Altitude	−0.13 ± 0.04	−3.00	**.0046**	**.024**	−0.10 ± 0.05	−1.92	.055	$\chi_{5}^{2}$ = 8.63, *p* = .12
Latitude	0.62 ± 0.32	1.94	.059	.18	−0.31 ± 0.70	−0.45	.65	
Longitude	0.14 ± 0.05	2.68	**.011**	**.040**	0.19 ± 0.12	1.60	.11	
Aspect (sin)	0.26 ± 0.25	1.06	.30	.56	0.24 ± 0.29	0.83	.41	
Aspect (cos)	0.01 ± 0.27	0.04	.97	.97	−0.10 ± 0.28	−0.37	.71	
Climatic parameters								
Climate PC1	0.00 ± 0.05	−0.07	.95	.97	−0.05 ± 0.05	−0.99	.32	$\chi_{4}^{2}$ = 7.00, *p* = .14
Climate PC2	−0.22 ± 0.06	−3.70	**.00065**	**.0098**	−0.23 ± 0.06	−3.58	**.00035**	
Climate PC3	−0.05 ± 0.06	−0.82	.42	.70	−0.03 ± 0.07	−0.44	.66	
Climate PC4	−0.01 ± 0.14	−0.09	.93	.97	−0.06 ± 0.13	−0.46	.64	
Habitat parameters								
Bare ground	−0.05 ± 0.03	−1.77	.08	.21	−0.05 ± 0.04	−1.28	.20	$\chi_{6}^{2}$ = 15.61, ***p*** ** = .016**
Shrub cover	0.00 ± 0.01	0.36	.72	.97	0.00 ± 0.02	−0.17	.86	
Stone cover	0.03 ± 0.02	1.66	.10	.22	0.04 ± 0.03	1.29	.20	
Vegetation cover	0.00 ± 0.01	−0.37	.72	.97	0.01 ± 0.02	0.26	.79	
Juniperus cover	0.00 ± 0.02	−0.06	.95	.97	0.02 ± 0.03	0.73	.47	
Grazing	0.80 ± 0.27	2.98	**.0049**	**.024**	0.90 ± 0.31	2.89	**.0038**	

The univariate block shows estimates from GLMMs controlled for site and region as random effects and, for models on green morphs, also for sex as a fixed factor, while other parameters were fitted one at a time. p* shows *p* value corrected for multiple testing. The multivariate block shows fits from models that included all predictors from each block simultaneously. Full model tests (FMTs) refer to full model tests within blocks. FMT across all variables was significant for both the occurrence of green morphs ($\chi_{15}^{2}$ = 30.44, *p* = .010) and the occurrence of pied morphs ($\chi_{15}^{2}$ = 30.00, *p* = .012), albeit model convergence was impaired. P‐values <0.05 are shown in bold.

**Table 2:** Loadings of original climatic predictors on the first four PC scores

Original BIOCLIM variable	PC1	PC2	PC3	PC4
BIO1 = annual mean temperature	−0.16	−0.24	−**0.36**	0.03
BIO2 = mean diurnal range	0.30	−0.08	−0.10	**0.37**
BIO3 = isothermality (BIO2/BIO7) (* 100)	0.11	0.02	−**0.31**	0.15
BIO4 = temperature seasonality (standard deviation *100)	0.28	−0.16	0.10	**0.33**
BIO5 = max temperature of warmest month	0.03	−0.27	−**0.36**	**0.31**
BIO6 = min temperature of coldest month	−0.27	−0.08	−0.29	−0.16
BIO7 = temperature annual range (BIO5‐BIO6)	**0.30**	−0.11	0.04	**0.39**
BIO8 = mean temperature of wettest quarter	0.11	−**0.41**	0.14	−0.13
BIO9 = mean temperature of driest quarter	−0.22	0.22	−0.25	0.00
BIO10 = mean temperature of warmest quarter	−0.09	−0.29	−**0.35**	0.12
BIO11 = mean temperature of coldest quarter	−0.24	−0.12	−**0.33**	−0.12
BIO12 = annual precipitation	−0.29	−0.14	0.19	0.18
BIO13 = precipitation of wettest month	−0.23	−**0.30**	0.19	0.02
BIO14 = precipitation of driest month	−**0.31**	0.09	0.12	**0.30**
BIO15 = precipitation seasonality (coefficient of variation)	−0.28	0.12	−**0.40**	0.03
BIO16 = precipitation of wettest quarter	−0.23	−0.29	0.21	0.04
BIO17 = precipitation of driest quarter	−**0.30**	0.06	0.16	**0.32**
BIO18 = precipitation of warmest quarter	−0.15	−**0.35**	0.25	−0.01
BIO19 = precipitation of coldest quarter	−**0.30**	0.15	0.05	**0.31**

Bold values indicate strong loadings (≥) of BIOCLIM variables with PC scores.

The proportion of pied individuals significantly decreased with altitude and increased with longitude, although these effects were not significant after multiple testing control (Table 1). Latitude and aspect did not significantly affect the proportion of pied individuals (Table 1). The proportion of pied individuals decreased with higher values of PC2 of the BIOCLIM variables (Table 1), and PC2 loadings were negatively with temperature and variable with precipitation tending to load negatively (Table 2), suggesting that the pied morphs decreased in frequency with lower temperatures and with lower precipitation during summer and the wettest month.

### 3.4 Habitat characteristics

The proportion of green individuals was not significantly influenced by shrub, stone, juniper, herbal and grassy vegetation cover, and grazing (Table 1). Bare ground cover tended to have a positive effect on the occurrence of green morphs, but the effect was not significant after multiple testing correction and also not in a multiple regression (Table 1). The proportion of pied individuals was not significantly influenced by bare ground, shrub, stone, juniper, and herbal and grassy vegetation cover (Table 1), but grazing had a significant positive effect on the proportion pied and this effect was significant after multiple testing correction and also in the multiple regression (Table 1).

### 3.5 Color variants

Front pattern was highly variable from being plain or plain with small black marks to being boldly marked black. Green individuals were typically largely unmarked, while brown individual were more variable. However, it was mostly pied females that showed strong, bold marks at their fronts (Figure S1). A total of 176 individuals (7.9% of females, 0.5% of males) showed a clear pink color tinge (GLM for sex difference: b = −2.75 ± 0.41, z = −6.67, *p* < 10^−10^) that was most common among brown (5.4%) and pied (5.2%) individuals and almost absent among greens (0.5%) (GLM for morph differences green vs. brown/pied: b = −2.48 ± 0.62, z = −3.97, *p* < 10^−4^). There was no evidence for an altitudinal trend in the occurrence of pinkish individuals (GLM: b = 0.062 ± 0.040, z = 1.58, *p* = .11).

### 3.6 Deformations and infections

While scoring color morphs, we also recorded occasional deformations. A total of 165 individuals had missing hind legs (5.0% of females, 2.8% of males), and 26 individuals (0.6% in females, 0.6% in males) had deformed or damaged wings. A total of 346 individuals carried ectoparasitic mites (9.5% in females, 10.3% in males, 26.8% in green, 66.2% in brown, and 7.1% in pied individuals), with mite parasitism varying strongly among sites (23 sites without mite recordings and up to 56% at other sites). Mite infection rates significantly increased with altitude (GLMM: b = 0.0043 ± 0.0010, z = 4.20, *p* = .00014, Figure S2).

## 4 DISCUSSION

We here analyse the spatial distribution of one distinctive and one subtler color polymorphism in the club‐legged grasshopper across most of its range in the European Alps. All populations were green–brown polymorphic with the possible exception of two sites in the east of the range that possibly lack green morphs (although they may occur in low numbers). Pied morphs were also present at all sites alongside typical brown morphs. We found significant spatial heterogeneity in both polymorphisms, particularly among sites within regions, and relative temporal stability across two consecutive years in color morphs ratios (with some changes in pied morphs). Weather conditions and population sizes of *G. sibiricus* vary substantially across years (Illich & Windig, 1999), so that stronger fluctuations of morphs ratios might have been expected. Although longer time‐series are desired, the data are more suggestive of spatial rather than temporal variability.

We found evidence that the occurrence of green and pied morphs depends on identifiable climatic conditions, while the abundance of pied morphs was also influenced by habitat characteristics. These findings argue for a role of selection in shaping local morph ratios. Furthermore, we found substantial unexplained variability among sites in morph ratios, suggesting a role of spatially heterogeneous selection caused by unexplored environmental conditions and/or genetic drift. In combination, the rather high frequency of all three color morphs, the evidence for environment dependence of color morph ratios and the reasoning that genetic drift alone is unlikely to maintain populations polymorphic in the long run (Gray & McKinnon, 2007; Svensson & Abbott, 2005) suggest that the most likely explanation for the maintenance of the color polymorphisms involves small‐scale migration–selection balance or local balancing (in particular negative frequency‐dependent) selection.

Which mechanism may ensure small‐scale heterogeneous selection to maintain populations polymorphic in selection–migration balance? Mountains are characterized by pronounced gradients with respect to altitude and aspect that, in combination with geomorphological and topological heterogeneity, harbor large variety of habitats even at a small spatial scale (Nagy & Grabherr, 2009). If mainly geographic, relatively large‐scale gradients such as altitude and aspect were the main drivers of color morph composition, we would expect to find strong correlations (as are indeed found in alpine populations of the meadow grasshoppers *Chorthippus parallelus*, Köhler, Samietz, & Schielzeth, 2017). Instead, the observed pattern of color morph composition varies at smaller scales in the range of a few hundred meters to some kilometers that matches habitat variability and also the likely dispersal range of the species (see Ingrisch & Köhler, 1998 for an overview of mobility data for various European grasshoppers). The small‐scale heterogeneity may result in spatially heterogeneous selection regimes in local populations that are connected by gene flow. Our data are thus consistent with migration–selection balance that involves a component of selection indicated by spatial heterogeneity and environmental correlates and migration indicated by the small spatial scale of color morph variation.

Migration–selection balance can maintain polymorphisms if the selection regime is spatially heterogeneous. Alternatively or additionally, polymorphisms may also be maintained locally by negative frequency‐dependent selection, a form of temporal fluctuation in selection that systematically favors rare morphs. What are the selective agents that may impose negative frequency‐dependent selection or selective trade‐offs? Visual predators are candidate agents of negative frequency‐dependent selection if they develop search images for the most abundant color morph (Bond & Kamil, 1998; Dukas, 2002; Punzalan, Rodd, & Hughes, 2005). Passerine birds, lizards, and frogs are abundant predators in the habitats of the club‐legged grasshopper, but there is currently no data on search image formation among potential predators of grasshoppers in the European Alps. We would expect such visual predators to be sensitive to color morph compositions across the community of grasshoppers. Among the most abundant grasshoppers in the same habitats are two brown/nongreen (*Podisma pedestris* and *Chorthippus brunneus*), one green (*Euthystira brachyptera*), and five polymorphic (*Melanoplus frigidus*,* Arcyptera fusca*,* Omocesthus viridulus*,* Omocesthus haemorrhoidalis, Stenobothrus lineatus*, and *Chorthippus parallelus*) species. It is currently unknown whether the color morph composition of the grasshopper community is related to color morph variation in individual species, but by the above reasoning, differences in community composition may contribute to spatial variability in morph ratios.

Migration–selection balance and frequency‐dependent selection ultimately require the existence of multiple fitness peaks either in space (as for migration–selection balance) or in time (as for negative frequency‐dependent selection). The two mechanisms are not mutually exclusive and may act in combination with other forms of multimodal fitness peaks also contributing to the maintenance of the polymorphism. Thermoregulation, predator avoidance behavior, parasite infestation, but also social interactions and mate choice can induce selective trade‐offs across contexts that can create multimodality in the fitness landscape with discrete alternative peaks (Ahnesjö & Forsman, 2006; Forsman, 2000; Karpestam, Wennersten, & Forsman, 2012). Multimodal fitness peaks may be reinforced when individuals seek out matching (micro)habitats (Edelaar, Siepielski, & Clobert, 2008; Wennersten & Forsman, 2012; Wennersten, Karpestam, & Forsman, 2012), but so far we have no evidence that green, brown, and pied individuals choose different microhabitats.

Beyond the three discrete color morphs brown, pied, and green, we recorded occasional pink variants. Such variants are known to occur in other species of grasshoppers as well, usually at low frequencies, and the pink color is thought to be caused by reduced forms of insectorubin (Uvarov, 1966). Variation in “pinkishness” is gradual ranging from weak tinges of pink to intense purple, as opposed to the discrete nature of the three main color morphs. Pink and purple colors occur regularly among brown and pied individuals, but are rare among green morphs. Own observations from the laboratory suggest that once developed, pink/purple colors persist for life. Whether there is a genetic predisposition for the development of pink/purple colors is currently unclear.

In the present study, we aimed to characterize spatial variation in color morphs the in club‐legged grasshopper *G. sibiricius* to shed light on which mechanisms may be involved in maintaining populations polymorphic (Gosden, Stoks, & Svensson, 2011). Among 42 populations across the alpine range of the species, we found almost universal coexistence of three color morphs with significant spatial variation mostly on a small (hundreds of meters to kilometers), but not on a very fine spatial scale (of a few dozens of meters). This indicates a role for local adaption in shaping morph ratios, in particular as we could identify environmental variables that co‐vary with local color morph rations. Local adaptation in combination with gene flow offers a likely contribute to the maintenance of the polymorphism. This does not exclude a role, even a predominant role, of local balancing selection for example by negative frequency dependency of color morph‐specific fitness. Fitness assays are required to address the mode and role of selection in maintaining color polymorphisms in this species.

## CONFLICT OF INTEREST

None declared.

## AUTHOR CONTRIBUTIONS

HS conceived the study. All authors contributed to study design and data collection. PD and HS analyzed the data. PD led the writing of the manuscript. All authors contributed to manuscript revision.

## DATA ACCESSIBILITY

Data are available from the Dryad Digital Repository: https://doi.org/10.5061/dryad.676c7g5.

## References

[ece34156-bib-0001] Ahnesjö, J. , & Forsman, A. (2006). Differential habitat selection by pygmy grasshopper color morphs; interactive effects of temperature and predator avoidance. Evolutionary Ecology, 20, 235–257. 10.1007/s10682-006-6178-8

[ece34156-bib-0002] Bates, D. , Mächler, M. , Bolker, B. , & Walker, S. (2015). Fitting linear mixed‐effects models using lme4. Journal of Statistical Software, 67, 1–48. 10.18637/jss.v067.i01

[ece34156-bib-0003] Behe, M. J. (2010). Experimental evolution, loss‐of‐function mutations, and “the first rule of adaptive evolution”. The Quarterly Review of Biology, 85, 419–445. 10.1086/656902 21243963

[ece34156-bib-0004] Bell, G. (2010). Fluctuating selection: The perpetual renewal of adaptation in variable environments. Philosophical Transactions of the Royal Society of London. Series B, Biological Sciences, 365, 87–97. 10.1098/rstb.2009.0150 20008388 PMC2842698

[ece34156-bib-0005] Bellmann, H. (2006). Der Kosmos Heuschreckenführer. Stuttgart, Germany: Franckh‐Kosmos.

[ece34156-bib-0006] Bellmann, H. , & Luquet, C. H. (2009). Guide des sauterelles, grillons et criquets d'Europe occidentale. Paris, France: Delachaux et Niestlé.

[ece34156-bib-0007] Benjamini, Y. , & Hochberg, Y. (1995). Controlling the false discovery rate: A practical and powerful approach to multiple testing. Journal of the Royal Statistical Society B, 57, 289–300.

[ece34156-bib-0008] Bond, A. B. , & Kamil, A. C. (1998). Apostatic selection by blue jays produces balanced polymorphism in virtual prey. Nature, 395, 594–596. 10.1038/26961

[ece34156-bib-0009] Bürger, R. , & Lande, R. (1994). On the distribution of the mean and variance of a quantitative trait under mutation‐selection‐drift balance. Genetics, 138, 901–912. 7851784 10.1093/genetics/138.3.901 PMC1206237

[ece34156-bib-0010] Bürger, R. , Wagner, G. P. , & Stettinger, F. (1989). How much heritable variation can be maintained in finite populations by mutation selection balance. Evolution, 43, 1748–1766. 10.1111/j.1558-5646.1989.tb02624.x 28564325

[ece34156-bib-0011] Comeault, A. A. , Carvalho, C. F. , Dennis, S. , Soria‐Carrasco, V. , & Nosil, P. (2016). Color phenotypes are under similar genetic control in two distantly related species of Timema stick insect. Evolution, 70, 1283–1296. 10.1111/evo.12931 27130287

[ece34156-bib-0012] Core Team, R. (2016). R: A language and environment for statistical computing. Vienna, Austria: R Foundation for Statistical Computing.

[ece34156-bib-0013] Dearn, J. M. (1990). Color pattern polymorphism In Chapman R. F., & Joern A. (Eds.), Biology of grasshoppers (pp. 517–549). New York, NY: John Wiley & Sons.

[ece34156-bib-0014] Dukas, R. (2002). Behavioural and ecological consequences of limited attention. Philosophical Transactions of the Royal Society of London. Series B, Biological Sciences, 357, 1539–1547. 10.1098/rstb.2002.1063 12495511 PMC1693070

[ece34156-bib-0015] Edelaar, P. , Siepielski, A. M. , & Clobert, J. (2008). Matching habitat choice causes directed gene flow: A neglected dimension in evolution and ecology. Evolution, 62, 2462–2472. 10.1111/j.1558-5646.2008.00459.x 18637835

[ece34156-bib-0016] Fisher, R. A. (1930). The evolution of dominance in certain polymorphic species. American Naturalist, 64, 385–406. 10.1086/280325

[ece34156-bib-0017] Fitzpatrick, M. J. , Feder, E. , Rowe, L. , & Sokolowski, M. B. (2007). Maintaining a behaviour polymorphism by frequency‐dependent selection on a single gene. Nature, 447, 210–212. 10.1038/nature05764 17495926

[ece34156-bib-0018] Ford, E. B. (1965). Genetic polymorphism. London, UK: Faber & Faber.

[ece34156-bib-0019] Forsman, A. (2000). Some like it hot: Intra‐population variation in behavioral thermoregulation in color‐polymorphic pygmy grasshoppers. Evolutionary Ecology, 14, 25–38. 10.1023/A:1011024320725

[ece34156-bib-0020] Forstmeier, W. , & Schielzeth, H. (2011). Cryptic multiple hypotheses testing in linear models: Overestimated effect sizes and the winner's curse. Behavioral Ecology and Sociobiology, 65, 47–55. 10.1007/s00265-010-1038-5 21297852 PMC3015194

[ece34156-bib-0021] Fuzeau‐Braesch, S. (1972). Pigments and color changes. Annual Review of Entomology, 17, 403–424. 10.1146/annurev.en.17.010172.002155

[ece34156-bib-0022] Gigord, L. D. B. , Macnair, M. R. , & Smithson, A. (2001). Negative frequency‐dependent selection maintains a dramatic flower color polymorphism in the rewardless orchid *Dactylorhiza sambucina* (L.) Soo. Proceedings of the National Academy of Sciences of the United States of America, 98, 6253–6255. 10.1073/pnas.111162598 11353863 PMC33454

[ece34156-bib-0023] Gosden, T. P. , Stoks, R. , & Svensson, E. I. (2011). Range limits, large‐scale biogeographic variation, and localized evolutionary dynamics in a polymorphic damselfly. Biological Journal of the Linnean Society, 102, 775–785. 10.1111/j.1095-8312.2011.01619.x

[ece34156-bib-0024] Gray, S. M. , & McKinnon, J. S. (2007). Linking color polymorphism maintenance and speciation. Trends in Ecology & Evolution, 22, 71–79. 10.1016/j.tree.2006.10.005 17055107

[ece34156-bib-0025] Huxley, J. (1955). Morphism and evolution. Heredity, 9, 1–51. 10.1038/hdy.1955.1

[ece34156-bib-0026] Illich, I. P. , & Windig, N. (1999). Dynamik von Heuschrecken‐Populationen (Orthoptera: Saltoatoria) in subalpinen und alpinen Rasen des Nationalparks Hohe Tauern (Österreichische Zentralalpen) von 1990 bis 1997. Wissenschaftliche Mitteilungen aus dem Nationalpark Hohe Tauern, 5, 63–85.

[ece34156-bib-0027] Ingrisch, S. , & Köhler, G . (1998). Die Heuschrecken Mitteleuropas. Magdeburg, Germany: Westarp Wissenschaften Magdeburg.

[ece34156-bib-0028] Karger, D. , Conrad, O. , Böhner, J. , Kawohl, T. , Kreft, H. , Soria‐Auza, R. , … Kessler, M . (2017a). Data from: Climatologies at high resolution for the earth's land surface areas, Dryad Digital Repository. 10.5061/dryad.kd1d4 PMC5584396 28872642

[ece34156-bib-0029] Karger, D. N. , Conrad, O. , Böhner, J. , Kawohl, T. , Kreft, H. , Soria‐Auza, R. W. , … Kessler, M. (2017b). Climatologies at high resolution for the earth's land surface areas. Scientific Data, 4, 170122 10.1038/sdata.2017.122 28872642 PMC5584396

[ece34156-bib-0030] Karpestam, E. , Wennersten, L. , & Forsman, A. (2012). Matching habitat choice by experimentally mismatched phenotypes. Evolutionary Ecology, 26, 893–907. 10.1007/s10682-011-9530-6

[ece34156-bib-0031] Köhler, G. , Samietz, J. , & Schielzeth, H. (2017). Morphological and colour morph clines along an altitudinal gradient in the meadow grasshopper *Pseudochorthippus parallelus* . PLoS ONE, 12, e0189815 10.1371/journal.pone.0189815 29284051 PMC5746220

[ece34156-bib-0032] Leyer, I. , & Wesche, K. (2007). Multivariate Statistik in der Ökologie. Berlin, Germany: Springer Verlag.

[ece34156-bib-0033] Misof, B. , Liu, S. , Meusemann, K. , Peters, R. S. , Donath, A. , Mayer, C. , … Zhou, X. (2014). Phylogenomics resolves the timing and pattern of insect evolution. Science, 346, 763–767. 10.1126/science.1257570 25378627

[ece34156-bib-0034] Morrissey, M. B. , & Hadfield, J. D. (2012). Directional selection in temporally replicated studies is remarkably consistent. Evolution, 66, 435–442. 10.1111/j.1558-5646.2011.01444.x 22276539

[ece34156-bib-0035] Nagy, L. , & Grabherr, G . (2009). The biology of Alpine habitats. Oxford, UK: Oxford University Press.

[ece34156-bib-0036] Nakagawa, S. , & Schielzeth, H. (2010). Repeatability for Gaussian and non‐Gaussian data: A practical guide for biologists. Biological Reviews, 85, 935–956. 10.1111/j.1469-185X.2010.00141.x 20569253

[ece34156-bib-0037] Punzalan, D. , Rodd, F. H. , & Hughes, K. A. (2005). Perceptual processes and the maintenance of polymorphism through frequency‐dependent predation. Evolutionary Ecology, 19, 303–320. 10.1007/s10682-005-2777-z

[ece34156-bib-0038] Rowell, C. H. F. (1971). The variable coloration of the Acridoid grasshoppers. Advances in Insect Physiology, 8, 145–198.

[ece34156-bib-0039] Sasaki, A. , & Ellner, S. (1997). Quantitative genetic variance maintained by fluctuating selection with overlapping generations: Variance components and covariances. Evolution, 51, 682–696. 10.1111/j.1558-5646.1997.tb03652.x 28568569

[ece34156-bib-0040] Shamim, G. , Ranjan, S. K. , Pandey, D. M. , & Ramani, R. (2014). Biochemistry and biosynthesis of insect pigments. European Journal of Entomology, 111, 149–164. 10.14411/eje.2014.021

[ece34156-bib-0041] Siepielski, A. M. , DiBattista, J. D. , & Carlson, S. M. (2009). It's about time: The temporal dynamics of phenotypic selection in the wild. Ecology Letters, 12, 1261–1276. 10.1111/j.1461-0248.2009.01381.x 19740111

[ece34156-bib-0042] Song, H. (2011). Density‐dependent phase polyphenism in nonmodel locusts: A minireview. Psyche: A Journal of Entomology, 2011, 1–16. 10.1155/2011/741769

[ece34156-bib-0043] Stoffel, M. A. , Nakagawa, S. , & Schielzeth, H. (2017). rptR: Repeatability estimation and variance decomposition by generalized linear mixed‐effects models. Methods in Ecology and Evolution, 8, 1639–1644. 10.1111/2041-210X.12797

[ece34156-bib-0044] Svensson, E. I. (2017). Back to basics: Using colour polymorphisms to study evolutionary processes. Molecular Ecology, 26, 2204–2211. 10.1111/mec.14025 28099782

[ece34156-bib-0045] Svensson, E. I. , & Abbott, J. (2005). Evolutionary dynamics and population biology of a polymorphic insect. Journal of Evolutionary Biology, 18, 1503–1514. 10.1111/j.1420-9101.2005.00946.x 16313463

[ece34156-bib-0046] Tanaka, S. , Harano, K. , Nishide, Y. , & Sugahara, R. (2016). The mechanism controlling phenotypic plasticity of body color in the desert locust: Some recent progress. Current Opinion in Insect Science, 17, 10–15. 10.1016/j.cois.2016.05.011 27720068

[ece34156-bib-0047] Uvarov, B. P . (1966). Grasshoppers and locusts: I. Anatomy, physiology, development, phase polymorphism and introduction to taxonomy. Cambridge, UK: Cambridge University Press.

[ece34156-bib-0048] Valverde, J. P. , & Schielzeth, H. (2015). What triggers colour change? Effects of background colour and temperature on the development of an alpine grasshopper. BMC Evolutionary Biology, 15, 1–12. 10.1186/s12862-015-0419-9 26293296 PMC4546165

[ece34156-bib-0049] Wennersten, L. , & Forsman, A. (2012). Population‐level consequences of polymorphism, plasticity and randomized phenotype switching: A review of predictions. Biological Reviews, 87, 756–767. 10.1111/j.1469-185X.2012.00231.x 22540928

[ece34156-bib-0050] Wennersten, L. , Karpestam, E. , & Forsman, A. (2012). Phenotype manipulation influences microhabitat choice in pygmy grasshoppers. Current Zoology, 58, 392–400. 10.1093/czoolo/58.3.392

[ece34156-bib-0051] West Eberhard, M. J. (2003). Developmental plasticity and evolution. New York, NY: Oxford University Press.

[ece34156-bib-0052] World Meterological Organization (2008). Guide to meteorological instruments and methods of observation. (pp. 1–681). Geneva, Switzerland: World Meterological Organization.

[ece34156-bib-0053] Yeaman, S. , & Whitlock, M. C. (2011). The genetic architecture of adaptation under migration‐selection balance. Evolution, 65, 1897–1911. 10.1111/j.1558-5646.2011.01269.x 21729046

