# Supplementary material for: Spatial analyses of two color polymorphisms in an alpine grasshopper reveal a role of small‐scale heterogeneity
Source: Ecol Evol. 2018 Jun 27;8(15):7273–84. doi: 10.1002/ece3.4156 (PMC6106198; doi:10.1002/ece3.4156)
Supplement: Supplementary file 1 [file ECE3-8-7273-s001.docx]

**Spatial analyses of two colour polymorphisms in an alpine grasshopper reveal a role of small-scale heterogeneity**

Petra Dieker^1,2^, Luisa Beckmann^1^, Julia Teckentrup^1^ & Holger Schielzeth^1,2^

^1^Department of Evolutionary Biology, Bielefeld University, Morgenbreede 45, 33615 Bielefeld, Germany

^2^ Department of Population Ecology, Institute of Ecology and Evolution, Friedrich Schiller University Jena, Dornburger Straße 159, 07743 Jena, Germany

# Supporting Information

**Table S1:** Overview of sampling sites and dates, and sample sizes (N). Country: CH Switzerland, A Austria.

| Country-canton/state | Region | Site name | Latitude (°N) | Longitude (°E) | Sampling date | N |
| --- | --- | --- | --- | --- | --- | --- |
|  |  |  |  |  |  |  |
| CH-Valais | R01 | Derborence | 46.273 | 7.210985 | 28/07/2014 | 61 |
| CH-Valais | R01 | Crans-Montana - Cry d'Err | 46.336 | 7.481776 | 25/07/2014 | 147 |
| CH-Valais | R01 | Crans-Montana - in direction Les Violettes | 46.327 | 7.491467 | 25/07/2014 | 119 |
| CH-Valais | R01 | Crans-Montana - Les Violettes | 46.341 | 7.498748 | 25/07/2014 | 105 |
| CH-Valais | R01 | Torrent-Alp | 46.356 | 7.646620 | 27/07/2014 | 154 |
| CH-Valais | R01 | Torrent-Alp/Mountain station Rinderhütte | 46.370 | 7.650307 | 31/07/2014 | 114 |
| CH-Valais | R01 | Torrent-Alp/Rinderhütte | 46.359 | 7.652634 | 27/07/2014 | 133 |
| CH-Valais | R01 | Fafleralp | 46.436 | 7.847391 | 24/07/2014 | 109 |
| CH-Valais | R02 | Upper Laggin Valley | 46.157 | 8.056610 | 30/07/2014 | 119 |
| CH-Valais | R02 | Lower Laggin Valley | 46.166 | 8.065235 | 30/07/2014 | 121 |
| CH-Valais | R02 | Egga | 46.203 | 8.107659 | 30/07/2014 | 154 |
| CH-Grisons | R03 | Oberalppass/Canals | 46.651 | 8.686650 | 28/07/2015 | 60 |
| CH-Grisons | R03 | Sedrun/Tgom | 46.665 | 8.766530 | 27/07/2015 | 101 |
| CH-Grisons | R04 | Julier Pass | 46.471 | 9.756150 | 08/03/2015 | 106 |
| CH-Grisons | R04 | Corviglia-Marguns-Piz Nair 1 | 46.516 | 9.772930 | 08/02/2015 | 103 |
| CH-Grisons | R04 | Corviglia-Marguns-Piz Nair 2 | 46.515 | 9.823280 | 08/02/2015 | 23 |
| CH-Grisons | R05 | Samedan/ Lower Muottas Muragl | 46.526 | 9.902170 | 08/04/2015 | 109 |
| CH-Grisons | R05 | Samedan/ Upper Muottas Muragl | 46.518 | 9.916080 | 08/04/2015 | 100 |
| CH-Grisons | R05 | Pontresina/Alp Languard | 46.488 | 9.923000 | 08/03/2015 | 101 |
| CH-Grisons | R05 | Bernina Pass | 46.443 | 9.980270 | 08/02/2015 | 68 |
| A-Vorarlberg | R06 | below Silvretta Bielerhöhe | 46.921 | 10.06820 | 22/08/2014 | 100 |
| **Continuation Table S1** | |  |  |  |  |  |
| Country-canton/state | Region | Site name | Latitude (°N) | Longitude (°E) | Sampling date | N |
|  |  |  |  |  |  |  |
| A-Tyrol | R06 | Silvretta Bielerhöhe | 46.920 | 10.09921 | 22/08/2014 | 21 |
| A-Tyrol | R06 | Birkhahnkopf/ Wirl | 46.963 | 10.14391 | 22/08/2014 | 50 |
| CH-Grisons | R07 | Fuorn Pass | 46.639 | 10.29060 | 08/05/2015 | 86 |
| A-Tyrol | R08 | Gaislachkogel/ Sölden | 46.952 | 10.98466 | 19/08/2014 | 16 |
| A-Tyrol | R09 | Timmelsjoch | 46.917 | 11.07898 | 20/08/2014 | 53 |
| A-Tyrol | R10 | Mountain station Halsmarter/ Tulfes | 47.240 | 11.54243 | 18/08/2014 | 14 |
| A-Tyrol | R11 | Eggalm/ Finkenberg | 47.163 | 11.70770 | 17/08/2014 | 100 |
| A-Tyrol | R11 | Penkenjoch/ Finkenberg | 47.172 | 11.79398 | 17/08/2014 | 35 |
| A-Tyrol | R12 | Thurntaler Rast/ Sillian | 46.774 | 12.40163 | 14/08/2014 | 77 |
| A-Tyrol | R12 | Zettersfeld/ Hochstein | 46.824 | 12.69260 | 15/08/2014 | 77 |
| A-Carinthia | R13 | Böse Platte/ Heiligenblut | 47.065 | 12.77200 | 28/07/2014 | 62 |
| A-Carinthia | R13 | Albitzen I /Heiligenblut | 47.067 | 12.77800 | 29/08/2015 | 600 |
| A-Carinthia | R13 | Albitzen II/ Heiligenblut | 47.066 | 12.78056 | 08/06/2015 | 43 |
| A-Carinthia | R14 | Millstätter Alm | 46.842 | 13.59378 | 10/08/2014 | 53 |
| A-Carinthia | R14 | Millstätter Hütte | 46.849 | 13.59630 | 10/08/2014 | 75 |
| A-Carinthia | R14 | Millstätter Törl | 46.850 | 13.59665 | 10/08/2014 | 70 |
| A-Styria | R15 | Ochsenkaralm/Bergbau | 47.449 | 14.42128 | 07/08/2014 | 10 |
| A-Styria | R16 | Mödlingerhütte/ Treffner Alm | 47.528 | 14.55353 | 08/08/2014 | 58 |
| A-Styria | R16 | Rotgraben/ Tonner Alm | 47.525 | 14.56530 | 08/08/2014 | 77 |
| A-Styria | R16 | Spielkogel | 47.519 | 14.56530 | 08/08/2014 | 101 |
| A-Lower Austria | R17 | Mitterbach Gemeindealp | 47.811 | 15.24680 | 04/08/2014 | 78 |
|  |  |  |  |  |  |  |

**
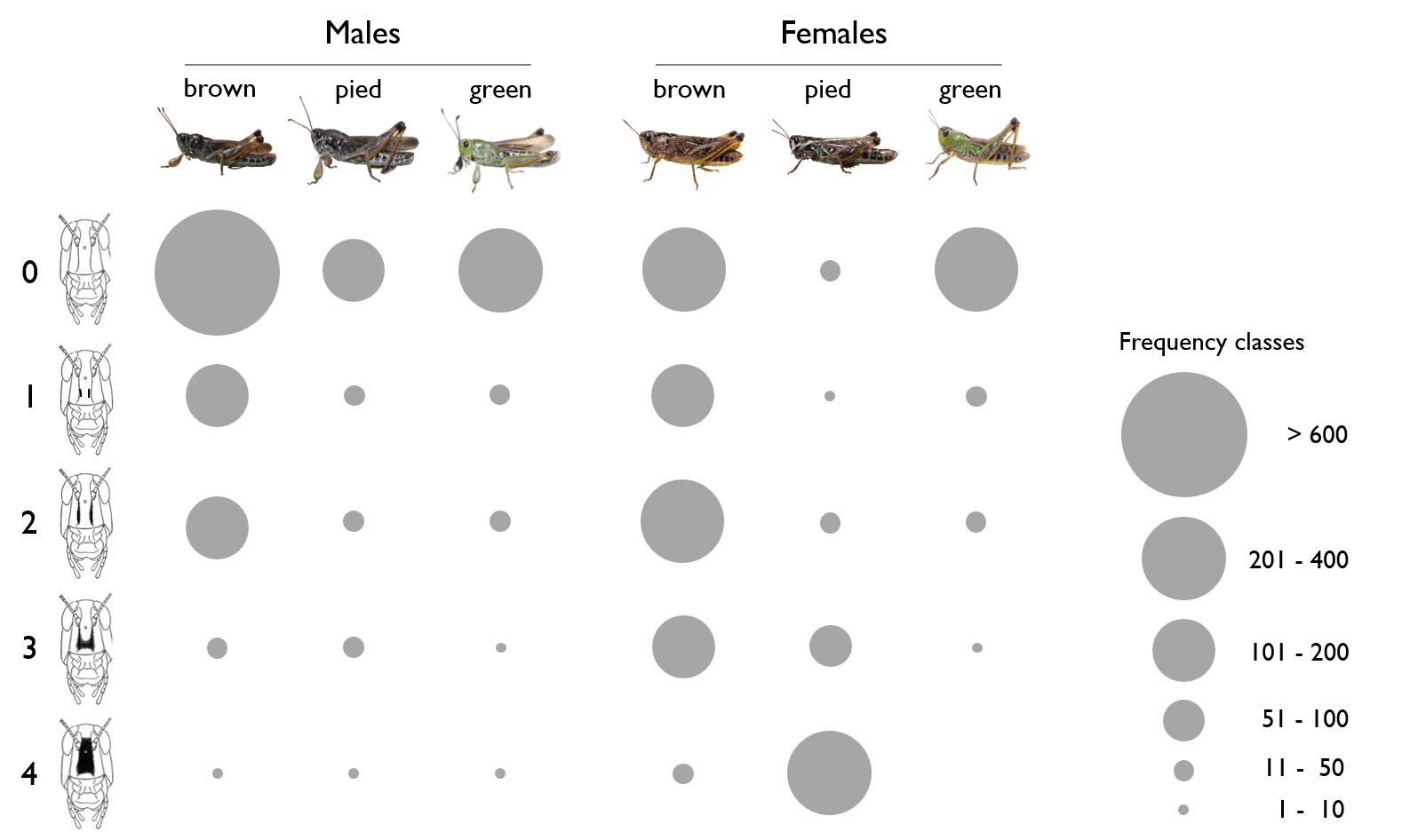
**

**Figure S1:** Variation among morphs in the pattern of the front. Front pattern was classified according to a 5-grade classification. Colour morph classification into brown, green and pied as done independent of the front pattern.

**
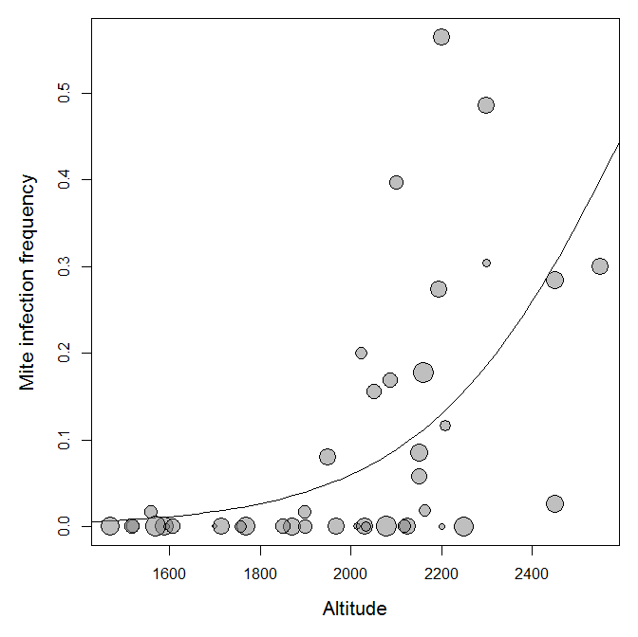
**

**Figure S2:** Proportion of individuals infected by ectoparasitic mites across all 42 sampling sites with the size of the dots proportional to the square root of the sample size.
